# NHAMCS Validation of Emergency Severity Index as an Indicator of Emergency Department Resource Utilization

**DOI:** 10.5811/westjem.2018.7.37556

**Published:** 2018-08-08

**Authors:** Michael B. Hocker, Charles J. Gerardo, B. Jason Theiling, John Villani, Rebecca Donohoe, Hirsh Sandesara, Alexander T. Limkakeng

**Affiliations:** * Medical College of Georgia, Augusta University, Department of Emergency Medicine and Hospitalist Services, Augusta, Georgia; † Duke University School of Medicine, Department of Emergency Medicine, Durham, North Carolina; ‡ Duke University, Durham Veterans Affairs Medical Center, Department of Emergency Medicine, Durham, North Carolina

## Abstract

**Introduction:**

Triage systems play a vital role in emergency department (ED) operations and can determine how well a given ED serves its local population. We sought to describe ED utilization patterns for different triage levels using the National Hospital Ambulatory Medical Care Survey (NHAMCS) database.

**Methods:**

We conducted a multi-year secondary analysis of the NHAMCS database from 2009-2011. National visit estimates were made using standard methods in Analytics Software and Solutions (SAS, Cary, NC). We compared patients in the mid-urgency range in regard to ED lengths of stay, hospital admission rates, and numbers of tests and procedures in comparison to lower or higher acuity levels.

**Results:**

We analyzed 100,962 emergency visits (representing 402,211,907 emergency visits nationwide). In 2011, patients classified as triage levels 1–3 had a higher number of diagnoses (5.5, 5.6 and 4.2, respectively) when compared to those classified as levels 4 and 5 (1.61 and 1.25). This group also underwent a higher number of procedures (1.0, 0.8 and 0.7, versus 0.4 and 0.4), had a higher ED length of stay (220, 280 and 237, vs. 157 and 135), and admission rates (32.2%, 32.3% and 15.5%, vs. 3.1% and 3.6%).

**Conclusion:**

Patients classified as mid-level (3) triage urgency require more resources and have higher indicators of acuity as those in triage levels 4 and 5. These patients’ indicators are more similar to those classified as triage levels 1 and 2.

## INTRODUCTION

The emergency department (ED) plays a pivotal role in providing healthcare for the nation, with the number of patients seeking care in EDs being estimated at 136 million per year.^1^ In addition, EDs often see more patients in a given time period than they have resources to provide care.^2^–^5^ In response to this issue, triage systems have been implemented to prioritize and allocate patients for these scarce resources.^6^–^10^ Given this vital role, initial triage designation can have a significant impact on any given patient’s experience in times of emergency illness. Although some triage systems have been proposed, it is currently unclear how well such systems perform to differentiate resource needs on a national scale.

Ranking ED patients based on perceived acuity of the illness or injury is necessary so that priorities can be established.^11^–^13^ Triage systems such as the Emergency Severity Index (ESI) are an important tool to accomplish this in EDs around the world. The current version of the ESI ranks acuity using five levels: (1) = Immediate or resuscitation, (2) = Very urgent, (3) = Urgent, (4) = Less urgent and (5) = Non-urgent.^14^ This five-level ESI has been validated across many metrics. Most EDs allocate dedicated spaces for patients at both ends of the spectrum of acuity: resuscitation and high-acuity care spaces often are used for patients triaged as levels 1 and 2, and “fast-track” spaces often are used for low-acuity patients triaged as levels 4 and 5. Despite these previous validation studies, it is currently unclear whether the ability of ESI triage levels to discriminate across resource utilization has been sustained over time, especially in the face of changes in patient sociodemographic, economic, clinical characteristics and crowded ED conditions. Furthermore, their performance has not been studied at a national level under real-life conditions.

Our anecdotal experience suggested that patients assigned level 3 triage acuity are often too complicated to be seen in a fast-track area and are not viewed as sick enough to compete with higher acuity patients for available beds. Especially in times of crowding, any non-acute designated beds are full with higher acuity patients, or admitted patients waiting for an inpatient bed. In addition, resource needs for level 3 triage patients seemed to be more similar to more-emergent, triage acuity levels despite having long wait times to see a physician and long overall ED lengths of stay (LOS).

Our objective was to compare patient sociodemographic, clinical characteristics, and utilization patterns for patients assigned different triage levels in the National Hospital Ambulatory Medical Care Survey (NHAMCS) database. We hypothesized that triage level 3 patients would require significantly more resources than levels 4 and 5 patients despite having similar wait times.

## METHODS

### Study Design and Setting

We conducted a secondary analysis of NHAMCS to compare patient sociodemographic, as well as clinical and utilization patterns, at different triage levels with a particular focus on triage level 3 (urgent) patients compared to other groups. Since the database shifted to a five-level triage system in the acuity-level classification in this database, we used the data from 2009–2011. We examined the effect of the potential changes in triage distributions during this period. This study is described per the Strengthening the Reporting of Observational studies in Epidemiology (STROBE) guidelines.^15^

### Ethics

This study was exempted from full review by our institutional review board.

### Database

We obtained the data from NHAMCS, a nationally representative survey conducted annually in the United States by the National Center for Health Statistics at the Centers for Disease Control and Prevention. We included datasets for the years 2009 through 2011 for an evaluation of triage-level categories. Data were collected on visits to outpatient and EDs of non-institutional, short-stay, and general hospitals located in 50 states and the District of Columbia, excluding federal, military, and Veterans Affairs hospitals.

**Population Health Research Capsule**

What do we already know about this issue?

Triage is a vital activity in the modern emergency department (ED). There are many systems for conducting triage in the ED that have been well validated.

What was the research question?

We sought to determine whether a patient’s triage classification accurately predicts subsequent resource utilization.

What was the major finding of the study?

Patients classified as mid-level (3) triage urgency require more resources and have higher indicators of acuity lower levels.

How does this improve population health?

More accurate understanding and prediction from triage can allow EDs to better assist the populations they serve.

NHAMCS uses a four-stage probability sampling design including selection of primary sampling units (PSU), hospitals within PSUs, clinics within hospitals, and patient visits within clinics. The exact methods of the NHAMCS survey have been described elsewhere.^16^ Briefly, hospitals are selected based on 112 geographic PSUs from the 1985–1994 National Health Interview Surveys. Approximately 480 hospitals within PSUs were surveyed. For the years included, an average of 411 hospitals were eligible, and an average of 369 participated for an unweighted average hospital sampling response rate of 89.8% annually. These hospitals are randomly assigned to 16 data collection groups that rotate across 13 four-week reporting periods throughout the year.

NHAMCS contractors (SRA International, Inc., Durham, NC) collect data from ED-visit medical records during a randomly assigned four-week period while being monitored by NHAMCS field representatives. NHAMCS staff members independently check 10% of the data for accuracy. Error rates are 0.3%–0.9% for various items on the survey. The NHAMCS survey records demographic data, payment source, provider types, procedures, prescriptions, laboratory and radiographic tests ordered for each visit, up to three reasons for visit (chief complaints), the ED diagnosis (*International Classification of Diseases Ninth Revision, Clinical Modification* (ICD-9-CM) codes), and the final hospital discharge diagnosis for those patients admitted to the hospital.^17^.

### Selection of Participants

The study sample includes all patients having a visit record to EDs in the NHAMCS.

### Variables

Our main variables of interest were triage level (immediacy with which patient should be seen, categorized as 1-Immediate; 2-Emergent; 3-Urgent; 4-Semi-urgent; 5-Non-urgent, and emergency service area does not conduct nursing triage); chief complaints for visit; primary diagnosis related to visit; total number of procedures provided; total number of tests/services provided; number of medications given in the ED; and visit disposition.

### Analysis

Our exploratory analysis started by evaluating distributions, frequencies, and percentages for each of the numeric and categorical variables. Categorical variables were evaluated for near-zero variation.^18^ We used graphical displays for both univariate analysis and bivariate associations. Missing data were explored using a combination of graphical displays involving univariate, bivariate, and multivariate methods. Imputation was performed using a k-nearest neighbors algorithm (n = 5).^19^ We generated population estimates through masked sample design variables, clustered PSUs, marker, and clustered PSUs, stratum marker, along with patient weights. We made use of line plots with confidence bands calculated to represent inferences to the U.S. population. All calculations were performed using the R language^20^ along with the survey package.^21^ Comparisons between the aggregate of triage level groups 1–3 vs. aggregate of 4–5 were made using Student’s t-test.

## RESULTS

We analyzed 100,962 emergency visits between 2009 and 2011, corresponding to 402,211,907 emergency visits when inferences were made to the entire U.S. population. Level 3 (Urgent) visits were the most frequent. The frequency of triage levels was stable over this period (Figure).

A total of 136,296,400 visits were inferred for 2011 in our analysis. Table 1 compares the five different acuity levels. Most patients in our sample were female (54.7%), except in the triage level 1 group. Level 3 was the most frequent triage acuity level, representing 42.3% of all cases. Patients triaged as levels 1–3 had a mean age above 40 years, while most patients in levels 4 and under were in their early thirties. There were significant differences in vital sign measures, pain level, reason for visit, and diagnoses across different triage acuity levels. The most common reasons for an ED visit were abdominal pain (8.1%), trauma (5.3%) and chest pain (5.2%). The most frequent diagnoses were trauma (18.1%), altered mental status (5.1%) and non-specified chest pain (3.6%).

In 2011, patients classified as levels 1–3 received a higher number of diagnoses (5.5, 5.6 and 4.2, respectively) when compared to those classified as semi-urgent and non-urgent (1.61 and 1.25) (Table 2). This group also underwent a higher number of procedures (1.0, 0.8 and 0.7, vs. 0.4 and 0.4), had a higher ED LOS (220, 280 and 237 minutes, vs. 157 and 135 minutes), and had higher admission rates (32.2%, 32.3% and 15.5%, vs. 3.1% and 3.6%). Finally, the level 1–3 group was also more frequently transferred (5.2%, 2.3% and 1.9%) when compared to the less-urgent group (below 0.5%). As expected, triage level 1 patients presented a markedly higher mortality rate (3.9%) when compared to other acuity levels. (See Appendix Tables 1–4.)

In assessing the impact of missing data, our imputation algorithms followed by sensitivity analyses did not demonstrate any directional changes in final conclusions.

## DISCUSSION

To the best of our knowledge, this is the first study evaluating ED triage acuity systems in the U.S., and their relationship to resource utilization. Triage systems have been extensively studied for validity. For example, it has been demonstrated that five levels are more reliable than a simpler system involving only three levels.^22^ In addition, ESI levels have been demonstrated to predict outcomes including hospital admission, length of hospital stay, and mortality rates.^23^,^24^ From 2009 to 2011, the NHAMCS classification was changed to a five-level system mirroring ESI, and the most common acuity level during this period was triage level 3 (level 3).

When evaluating 2011, patients classified as triage level 1, triage level 2, and triage level 3 formed a cluster that was consistent across sociodemographic, clinical and resource utilization categories, clearly differentiating themselves from semi-urgent and non-urgent patients. We identified a trend toward increased similarity among triage level 1–3 patients concerning resource utilization. In fact, within that group one may actually conclude that patients with ESI 2 are more similar to ESI 3 patients than ESI 1. While the admission rates for ESI 2 are more similar to those with ESI 1 (Table 3), the actual resource utilization and total LOS are more similar between ESI 2 and ESI 3.

Moreover, clinically it is easier to discern between an ESI of 1 vs. 2 purely based on obvious acuity at time of presentation then it is to distinguish between an ESI 2 vs. 3. That is to say, it is easier to determine that a patient is in need of “immediate resuscitation” upon their presentation than to distinguish whether a patient needs “urgent” vs. “very urgent” evaluation and management. Additionally, despite different admission rates, ESI 2 and 3 patients had similar need for procedures and testing, highlighting the clinical ambiguity and higher cognitive burden for providers treating these patients.

These similarities are striking considering the different resources allocated to these distinct populations. For example, most EDs have dedicated resuscitation or acute rooms for highest acuity patients (usually ESI 1 and some ESI 2 patients). Similarly, most departments also assign space (fast track, minor care) to the lowest triage acuity patients (ESI 4–5). The ESI 3 patients are often viewed as too sick for the less-acute areas and not sick enough to compete for the more-acute areas. Only recently have departments proposed a mechanism such as “middle track” or “flexible fast track” areas and physician triage processes to address the needs of triage level 3 patients.^6^,^8^

Our analysis suggests that such interventions are warranted and worthy of further research. Moreover, based on LOS, resource utilization and potential provider-cognitive burden, it may actually be necessary to develop areas where ESI 2 and 3 patients would be cohorted and treated together. In addition, some EDs have developed “non-acute” rooms to accommodate the middle triage groups. However, in times of crowding and increased acuity being seen across the country, these beds are now filled with higher acuity patients, or admitted patients waiting for an inpatient bed.

In agreement with previous reports, our analysis contradicts the myth that emergency services are being proportionally dedicated to non-urgent patients.^25^–^27^ In fact, at least based on triage, non-urgent patients represented less than 10% of all patients seeking emergency care. These findings have important consequences since potential policies referring non-urgent patients to facilities other than the ED might not reduce ED crowding and boarding as much as expected.^28^,^29^ Instead, measures aimed at optimizing ED workflow might be more effective.^30^–^36^

Our results likely reflect a growing shift in population mix for individuals seeking care at the ED, with fewer patients now falling into a non-urgent triage category. At the same time, level 3 cases have become more complicated, often requiring extensive evaluations. With an aging population, admissions and resources used per patient will likely require an increase in ED capacity of approximately 10%, with an increase in admissions predicted at 23%.^37^

The intensive use of health resources by mid-level urgency patients has important implications for patient safety and resource allocation in EDs, as urgent patients compete for resources with triage level 1 and 2 patients.^38^,^39^ This shift in case mix is important when devising ED workflow, ensuring that patients are not exposed to additional risk due to ED overload.

## LIMITATIONS

Despite filling an important gap in the literature, our study does have limitations. First, we did not evaluate whether triage assignment was reliable or uniform over time. It is possible that, along with a shift in case mix, the classification criteria used by triage professionals might also have changed. The ESI system has good inter-rater reliability, but its performance over time has never been assessed.^40^,^41^ Second, studies based on administrative data are susceptible to biases during the data collection process, ultimately affecting our results. Nevertheless, the NHAMCS has been widely used to study nationwide ED processes, and the key variables (triage level, admission rates, ED LOS, tests and studies ordered) we studied were straightforward, standard information collected on most ED visits. Third, missing data were present, which might have biased our results. To minimize this limitation, we used imputation algorithms followed by sensitivity analyses to ensure that our final conclusions were valid under different assumptions.

## CONCLUSION

We found that patients classified as triage level 3 (Urgent) are now one of the major components in the case mix for EDs, and their resource utilization profile is similar to triage levels 1 and 2 patients. These findings have implications for triage algorithms, emergency resource allocation, and care coordination. Future studies should prospectively evaluate the impact of different triage algorithms among patients presenting to the ED, considering both clinical as well as public health perspectives.

## Supplementary Information



## Figures and Tables

**Figure:**
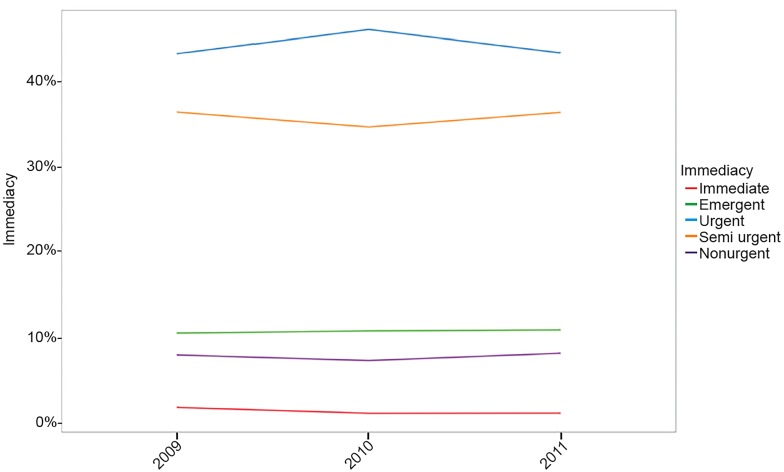
Frequency of emergency visits according to triage level between 2009 and 2011.

**Table 1:** Inferences for the United States regarding characteristics of study participants, chief complaints and primary diagnosis in 2011.

Variable	Total (136,296,400)	1-Immediate (1,638,167–1.2%)	2-Emergent (14,590,086–10.7%)	3-Urgent (57,689,765–42.3%)	4-Semi-urgent (48,452,556–35.5%)	5-Nonurgent (10,966,056–8.04%)	No triage (2,959,770)	P value
Patient age in years	37.14 (±0.46)	43.24 (±2.19)	46.02 (±0.96)	40.79 (±0.57)	31.73 (±0.57)	28.81 (±1.39)	38.10 (±1.6)	< .001
Female	74,620,514 (54.7% ±4.4%)	651,322 (39.8% ±8.2%)	7,547,970 (51.7% ±4.6%)	33,005,491 (57.2% ±3.7%)	26,106,889 (53.9% ±3.6%)	5,656,675 (51.6% ±6.2%)	1,652,167 (55.8% ±22%)	< .001
Hispanic ethnicity	15,932,426 (11.7% ±1.6%)	125,325 (7.7% ±2.2%)	1,570,681 (10.8% ±1.8%)	6,389,676 (11.1% ±1.2%)	5,983,797 (12.3% ±1.5%)	1,612,349 (14.7% ±2.5%)	250,598 (8.5% ±6.4%)	.318
Race								.268
Asian	2,248,640 (1.9% ±0.4%)	24,213 (1.7% ±0.9%)	262,507 (2% ±0.4%)	1,067,602 (2.2% ±0.4%)	722,486 (1.8% ±0.3%)	140,101 (1.5% ±0.3%)	31,731 (1.2% ±0.7%)	
Black	29,463,330 (25.4% ±3.5%)	270,646 (19.3% ±5%)	2,748,765 (21.5% ±3.1%)	12,217,466 (25% ±2.9%)	11,235,429 (27.6% ±3.5%)	2,563,547 (27% ±4.2%)	427,477 (15.6% ± 3.5%)	
Other	2,075,107 (1.8% ±0.6%)	27,202 (1.9% ±1.4%)	171,785 (1.3% ±0.4%)	779,101 (1.6% ±0.5%)	814,297 (2% ±0.5%)	115,022 (1.2% ±0.5%)	167,700 (6.1% ±5.2%)	
White	82,290,829 (70.9% ±6.4%)	1,078,307 (77% ±19.7%)	9,627,332 (75.2% ±6.8%)	34,778,809 (71.2% ± 4.7%)	28,004,392 (68.7% ±5.4%)	6,685,121 (70.3% ±10.1%)	2,116,868 (77.2% ±30.7%)	
Presenting level of pain	4.93 (±0.06)	3.93 (±0.22)	4.36 (±0.17)	5.00 (±0.07)	5.16 (±0.08)	4.39 (±0.17)	4.85 (±0.2)	< .001
Waiting time to see provider	48.89 (±2.37)	25.44 (±4.44)	40.21 (±3.7)	51.81 (±3.01)	51.32(± 2.65)	41.85 (±2.29)	33.92 (±11.47)	.005
Visit reason								
Abdominal pain	11,069,123 (8.1% ±0.9%)	30,554 (1.9% ±0.9%)	964,851 (6.6% ±1.2%)	7,336,403 (12.7% ±0.9%)	2,046,818 (4.2% ±0.5%)	406,777 (3.7% ±1%)	283,720 (9.6% ±5.1%)	< .001
Trauma	7,208,961 (5.3% ±0.6%)	147,099 (9% ±2.3%)	458,883 (3.1% ±0.4%)	2,150,809 (3.7% ±0.4%)	3,743,009 (7.7% ±0.7%)	554,235 (5.1% ±1%)	154,926 (5.2% ±2.3%)	< .001
Chest pain	7,051,953 (5.2% ±0.6%)	191,780 (11.7% ±4.3%)	2,152,744 (14.8% ±1.7%)	3,417,028 (5.9% ±0.5%)	966,750 (2% ±0.2%)	200,630 (1.8% ±0.5%)	123,021 (4.2% ±2.3%)	< .001
Diagnosis								
Trauma	24,806,511 (18.2% ±1.6%)	242,168 (14.8% ±3.3%)	1,322,862 (9.1% ±1.1%)	7,227,376 (12.5% ±0.9%)	13,150,153 (27.1% ±1.9%)	2,241,924 (20.4% ±3.2%)	622,028 (21% ±7.8%)	< .001
Altered mental status	6,999,248 (5.1% ±0.6%)	106,805 (6.5% ±2.2%)	1,122,289 (7.7% ±0.9%)	3,864,029 (6.7% ±0.6%)	1,412,586 (2.9% ±0.3%)	325,682 (3% ±0.6%)	167,857 (5.7% ±2.4%)	< .001
Non-specified chest pain	4,939,289 (3.6% ±0.5%)	117,538 (7.2% ±3%)	1,753,781 (12% ±1.6%)	2,455,365 (4.3% ±0.4%)	463,078 (1% ±0.1%)	87,980 (0.8% ±0.3%)	61,547 (2.1% ±0.9%)	< .001

**Table 2:** Resource utilization of emergency departments according to triage acuity level in 2011.

Variable	Total (136,296,400)	1-Immediate (1,638,167)	2-Emergent (14,590,086)	3-Urgent (57,689,765)	4-Semi-urgent (48,452,556)	5-Nonurgent (10,966,056)	No triage (2,959,770)	P value
Length of visit	203.59 (±4.81)	219.72 (±14.34)	280.11 (±10.5)	237.01 (±5.84)	157.36 (±4.21)	134.65 (±6.63)	167.65 (±26.86)	< .001
Number of procedures	0.57 (±0.02)	0.97 (±0.1)	0.77 (±0.03)	0.67 (±0.02)	0.44 (±0.02)	0.36 (±0.02)	0.51 (±0.06)	< .001
Number of diagnostics	3.18 (±0.1)	5.45 (±0.71)	5.56 (±0.21)	4.20 (±0.14)	1.61 (±0.08)	1.25 (±0.09)	3.01 (±0.45)	< .001
Number of medications given in ED	1.41 (±0.03)	2.31 (±0.2)	1.97 (±0.09)	1.64 (±0.04)	1.04 (±0.03)	0.95 (±0.08)	1.38 (±0.17)	< .001
Hospital admission	16,228,737 (11.9% ±1.3%)	527,876 (32.2% ±8.8%)	4,712,570 (32.3% ±3.3%)	8,942,106 (15.5% ±1.3%)	1,483,549 (3.1% ±0.5%)	394,351 (3.6% ±1%)	168,285 (5.7% ±3.3%)	< .001
Admission to observation unit, then hospitalized	964,907 (0.7% ±0.2%)	4,253 (0.3% ±0.2%)	286,256 (2% ±0.5%)	601,216 (1% ±0.2%)	65,924 (0.1% ±0%)	7,258 (0.1% ±0.1%)	0 (0% ±0%)	.024
Died in ED	143,498 (0.1% ±0%)	64,137 (3.9% ±1%)	26,907 (0.2% ±0.1%)	37,412 (0.1% ±0%)	3,252 (0% ±0%)	2,897 (0% ±0%)	8,893 (0.3% ±0.2%)	< .001
Transfer to other hospital	1,950,270 (1.4% ±0.3%)	84,900 (5.2% ±1.9%)	341,898 (2.3% ±0.4%)	1,088,620 (1.9% ±0.3%)	203,857 (0.4% ±0.1%)	55,836 (0.5% ±0.2%)	175,159 (5.9% ±2.4%)	< .001

*ED*, emergency department.

**Table 3:** Resource utilization according to acuity level in 2011 for Emergency Severity Index 2 vs. 3.

Variable	2-Emergent (14,590,086)	3-Urgent (57,689,765)
Length of visit	280.11 (±10.5)	237.01 (±5.84)
Number of procedures	0.77 (±0.03)	0.67 (±0.02)
Number of diagnostics	5.56 (±0.21)	4.20 (±0.14)
Number of medications given in ED	1.97 (±0.09)	1.64 (±0.04)
Return for appointment as needed	3,390,755 (23.2% ±3.2%)	18,584,580 (32.2% ±2.4%)
Return/refer to physician/ clinic for follow-up	7,333,338	37,836,752
	(50.3% ±5.2%)	(65.6% ±4.9%)
Hospital admission	4,712,570 (32.3% ±3.3%)	8,942,106 (15.5% ±1.3%)
Died in ED	26,907 (0.2% ±0.1%)	37,412 (0.1% ±0%)
Transfer to other hospital	341,898 (2.3% ±0.4%)	1,088,620 (1.9% ±0.3%)
	

*ED*, emergency department.
