# Supplementary material for: NHAMCS Validation of Emergency Severity Index as an Indicator of Emergency Department Resource Utilization
Source: West J Emerg Med. 2018 Aug 8;19(5):855–62. doi: 10.5811/westjem.2018.7.37556 (PMC6123086; doi:10.5811/westjem.2018.7.37556)
Supplement: Supplementary file 1 [file wjem-19-855-s001.docx]

**Appendix Table 1:** Inferences for the United States regarding characteristics of study participants, chief complaints and primary diagnosis in 2009.

| **Variable** | **Total**  (136,072,130) | **Immediate**  (2,493,833) | **Emergent**  (13,876,131) | **Urgent**  (56,654,302) | **SemiUrgent**  (47,766,622) | **Nonurgent**  (10,531,778) | **No** **triage** (4,749,464) | **p** |
| --- | --- | --- | --- | --- | --- | --- | --- | --- |
| Patient age in years | 36.27 (+- 0.52) | 41.35 (+- 1.74) | 44.82 (+- 0.87) | 39.78 (+- 0.47) | 31.00 (+- 0.86) | 29.06 (+- 1.31) | 35.77 (+- 2.11) | < .001 |
| Female | 74,538,836  (54.8% +- 4.8%) | 1,276,617  (51.2% +- 10.1%) | 7,409,715  (53.4% +- 4.6%) | 32,273,475  (57% +- 4.1%) | 25,578,107  (53.5% +- 4%) | 5,436,339  (51.6% +- 5.4%) | 2,564,583  (54% +- 17.6%) | < .001 |
| Hispanic ethnicity | 16,554,457  (12.2% +- 2%) | 242,220  (9.7% +- 2%) | 1,549,118  (11.2% +- 1.5%) | 6,783,637  (12% +- 1.4%) | 5,898,116  (12.3% +- 1.8%) | 1,344,437  (12.8% +- 2.1%) | 736,929  (15.5% +- 11.5%) | .668 |
| Race |  |  |  |  |  |  |  | .547 |
| Asian | 2,623,684  (2.2% +- 0.5%) | 32,253 (1.6% +- 0.7%) | 242,523  (1.9% +- 0.4%) | 1,294,682  (2.6% +- 0.6%) | 896,637  (2.1% +- 0.4%) | 139,295  (1.5% +- 0.3%) | 18,294  (0.5% +- 0.2%) |  |
| Black | 29,477,123  (24.2% +- 3.4%) | 356,860 (18% +- 3.8%) | 2,770,641  (21.9% +- 3%) | 12,016,103  (23.7% +- 2.6%) | 10,759,209  (25% +- 3.1%) | 2,549,696  (27.4% +- 5.1%) | 1,024,614  (26.2% +- 13.8%) |  |
| Other | 2,523,926  (2.1% +- 0.7%) | 72,792 (3.7% +- 1.6%) | 282,763  (2.2% +- 0.7%) | 1,061,692  (2.1% +- 0.6%) | 826,647  (1.9% +- 0.5%) | 128,929  (1.4% +- 0.5%) | 151,103  (3.9% +- 3.5%) |  |
| White | 86,971,415  (71.5% +- 6.6%) | 1,518,013  (76.7% +- 13.5%) | 9,340,646  (73.9% +- 7.2%) | 36,261,208  (71.6% +- 5.3%) | 30,629,809  (71% +- 6%) | 6,500,615  (69.8% +- 8.1%) | 2,721,124  (69.5% +- 22.9%) |  |
| Presenting level of pain | 4.87 (+- 0.07) | 4.50 (+- 0.19) | 4.28 (+- 0.12) | 4.95 (+- 0.08) | 5.08 (+- 0.1) | 4.20 (+- 0.2) | 5.02 (+- 0.42) | < .001 |
| Temperature in Fahrenheit | 98.30 (+- 0.03) | 98.26 (+- 0.1) | 98.20 (+- 0.03) | 98.29 (+- 0.02) | 98.33 (+- 0.04) | 98.30 (+- 0.1) | 98.29 (+- 0.11) | .336 |
| Heart rate | 92.64 (+- 0.49) | 92.94 (+- 1.6) | 92.79 (+- 0.68) | 91.53 (+- 0.38) | 93.69 (+- 0.81) | 93.86 (+- 1.44) | 92.11 (+- 1.44) | .028 |
| Blood pressure - Systolic | 132.69 (+- 0.43) | 130.26 (+- 1.58) | 136.51 (+- 0.91) | 133.96 (+- 0.39) | 130.77 (+- 0.76) | 130.01 (+- 1.34) | 130.92 (+- 1.54) | < .001 |
| Blood pressure - Diastolic | 77.30 (+- 0.27) | 75.33 (+- 1.3) | 78.39 (+- 0.5) | 77.59 (+- 0.27) | 76.86 (+- 0.35) | 76.76 (+- 0.61) | 76.75 (+- 0.68) | .011 |
| Respiratory rate | 19.87 (+- 0.12) | 20.28 (+- 0.41) | 20.38 (+- 0.15) | 19.71 (+- 0.1) | 19.82 (+- 0.19) | 20.22 (+- 0.32) | 20.03 (+- 0.2) | .027 |
| Pulse oximetry (percent) | 97.30 (+- 0.17) | 95.95 (+- 0.63) | 97.04 (+- 0.15) | 97.42 (+- 0.13) | 97.42 (+- 0.26) | 97.27 (+- 0.39) | 95.93 (+- 1.21) | .197 |
| Waiting time to see physician | 58.12 (+- 2.53) | 28.98 (+- 2.48) | 51.14 (+- 3.67) | 63.31 (+- 3.01) | 58.67 (+- 2.92) | 53.43 (+- 3.46) | 38.11 (+- 5.12) | < .001 |
| Visit reason |  |  |  |  |  |  |  |  |
| Shortness of breath | 3,710,119  (2.7% +- 0.3%) | 149,860  (6% +- 1.6%) | 806,728  (5.8% +- 0.6%) | 1,971,533  (3.5% +- 0.3%) | 557,686  (1.2% +- 0.2%) | 114,823  (1.1% +- 0.5%) | 109,489  (2.3% +- 0.8%) | < .001 |
| Chest pain | 7,169,155  (5.3% +- 0.6%) | 186,752  (7.5% +- 2.2%) | 1,988,644  (14.3% +- 1.8%) | 3,637,050  (6.4% +- 0.5%) | 971,785  (2% +- 0.2%) | 154,488  (1.5% +- 0.5%) | 230,436  (4.9% +- 1.8%) | < .001 |
| Abdominal pain | 9,597,271  (7.1% +- 0.8%) | 116,567  (4.7% +- 1.8%) | 619,796  (4.5% +- 0.5%) | 6,414,819  (11.3% +- 1%) | 1,922,602  (4% +- 0.5%) | 266,897  (2.5% +- 0.5%) | 256,590  (5.4% +- 1.7%) | < .001 |
| Dizziness | 1,780,155  (1.3% +- 0.2%) | 18,052  (0.7% +- 0.4%) | 182,781  (1.3% +- 0.3%) | 1,148,890  (2% +- 0.2%) | 331,163  (0.7% +- 0.1%) | 33,497(0.3% +- 0.1%) | 65,772  (1.4% +- 0.6%) | < .001 |
| Nausea | 4,816,124  (3.5% +- 0.4%) | 39,181  (1.6% +- 0.6%) | 349,414  (2.5% +- 0.4%) | 2,849,865  (5% +- 0.5%) | 1,183,333  (2.5% +- 0.3%) | 220,565  (2.1% +- 0.5%) | 173,766  (3.7% +- 1.2%) | < .001 |
| Shoulder pain | 1,311,893  (1% +- 0.2%) | 26,016  (1% +- 0.5%) | 79,438  (0.6% +- 0.1%) | 519,698  (0.9% +- 0.1%) | 579,858  (1.2% +- 0.1%) | 58,990  (0.6% +- 0.2%) | 47,893  (1% +- 0.4%) | .026 |
| Back pain | 3,696,122  (2.7% +- 0.3%) | 56,846  (2.3% +- 0.9%) | 175,805  (1.3% +- 0.2%) | 1,279,526  (2.3% +- 0.2%) | 1,773,116  (3.7% +- 0.4%) | 199,529  (1.9% +- 0.4%) | 211,300  (4.4% +- 1.7%) | < .001 |
| Diagnosis |  |  |  |  |  |  |  |  |
| Trauma | 7,106,240  (5.2% +- 0.6%) | 141,060  (5.7% +- 1.7%) | 436,346  (3.1% +- 0.6%) | 2,064,677  (3.6% +- 0.4%) | 3,539,175  (7.4% +- 0.7%) | 687,667  (6.5% +- 1.1%) | 237,315  (5% +- 2%) | < .001 |
| Fever | 7,373,097  (5.4% +- 0.7%) | 92,342  (3.7% +- 1.4%) | 488,084  (3.5% +- 0.5%) | 2,631,301  (4.6% +- 0.4%) | 3,285,521  (6.9% +- 0.9%) | 711,834  (6.8% +- 1.3%) | 164,015  (3.5% +- 1.6%) | < .001 |
| Cough | 4,684,301  (3.4% +- 0.5%) | 72,363  (2.9% +- 1.5%) | 228,489  (1.6% +- 0.3%) | 1,563,777  (2.8% +- 0.3%) | 2,291,095  (4.8% +- 0.5%) | 330,492  (3.1% +- 0.5%) | 198,085  (4.2% +- 1.6%) | < .001 |
| Nonspecified chest pain | 4,250,380  (3.1% +- 0.4%) | 108,496  (4.4% +- 1.4%) | 1,252,986  (9% +- 1.4%) | 2,257,593  (4% +- 0.4%) | 442,836  (0.9% +- 0.1%) | 48,878  (0.5% +- 0.1%) | 139,591  (2.9% +- 1.1%) | < .001 |
| Cardiac arrest and ventricular fibrillation | 139,791  (0.1% +- 0%) | 77,307  (3.1% +- 1%) | 28,228  (0.2% +- 0.1%) | 22,473  (0% +- 0%) | 8,635  (0% +- 0%) | 0  (0% +- 0%) | 3,148  (0.1% +- 0.1%) | < .001 |
| Arrhythmias | 787,652  (0.6% +- 0.1%) | 109,485  (4.4% +- 1.1%) | 345,480  (2.5% +- 0.4%) | 264,628  (0.5% +- 0.1%) | 51,513  (0.1% +- 0%) | 2,293  (0% +- 0%) | 14,253  (0.3% +- 0.1%) | < .001 |
| Congestive heart failure | 668,028  (0.5% +- 0.1%) | 32,941  (1.3% +- 0.5%) | 181,301 (1.3% +- 0.3%) | 383,417  (0.7% +- 0.1%) | 61,810  (0.1% +- 0%) | 0  (0% +- 0%) | 8,559  (0.2% +- 0.1%) | < .001 |
| Acute cerebrovascular disease | 1,822,719  (1.3% +- 0.2%) | 13,215  (0.5% +- 0.3%) | 365,704  (2.6% +- 0.4%) | 787,849  (1.4% +- 0.2%) | 460,362  (1% +- 0.3%) | 128,698  (1.2% +- 0.3%) | 66,891  (1.4% +- 0.6%) | < .001 |
| Syncope | 1,219,607  (0.9% +- 0.2%) | 31,352  (1.3% +- 0.5%) | 272,908  (2% +- 0.4%) | 699,189  (1.2% +- 0.1%) | 151,802  (0.3% +- 0.1%) | 24,219  (0.2% +- 0.1%) | 40,137  (0.8% +- 0.4%) | < .001 |
| Anxiety disorders | 1,025,072  (0.8% +- 0.2%) | 22,077  (0.9% +- 0.6%) | 111,329  (0.8% +- 0.2%) | 514,798  (0.9% +- 0.1%) | 279,763  (0.6% +- 0.1%) | 64,941  (0.6% +- 0.2%) | 32,164  (0.7% +- 0.3%) | .341 |
| Gastrointestinal disease | 16,126,931  (11.8% +- 1.3%) | 277,947  (11.1% +- 4.2%) | 1,136,688  (8.2% +- 0.9%) | 9,167,637  (16.2% +- 1.5%) | 4,126,572  (8.6% +- 0.8%) | 949,817  (9.1% +- 1.7%) | 468,270  (9.8% +- 3.2%) | < .001 |
| Altered mental status | 6,703,885  (4.9% +- 0.6%) | 111,362  (4.5% +- 1%) | 1,014,924  (7.3% +- 0.9%) | 3,583,101  (6.3% +- 0.5%) | 1,538,854  (3.2% +- 0.4%) | 311,428  (3% +- 0.8%) | 144,216  (3% +- 1%) | < .001 |
| Upper respiratory infections | 7,681,336  (5.6% +- 0.7%) | 96,178  (3.9% +- 1.3%) | 389,956  (2.8% +- 0.5%) | 2,270,397  (4% +- 0.4%) | 3,683,812  (7.7% +- 0.7%) | 886,853  (8.4% +- 1.1%) | 354,140  (7.5% +- 2.9%) | < .001 |
| Pneumonia | 3,060,497  (2.2% +- 0.4%) | 40,777  (1.6% +- 0.7%) | 339,739 (2.4% +- 0.4%) | 1,406,490  (2.5% +- 0.3%) | 959,785  (2% +- 0.3%) | 204,372  (1.9% +- 0.8%) | 109,334  (2.3% +- 0.8%) | .506 |
| Asthma/COPD | 4,343,772  (3.2% +- 0.4%) | 53,674  (2.2% +- 0.7%) | 617,737 (4.5% +- 0.6%) | 1,980,438  (3.5% +- 0.3%) | 1,258,740  (2.6% +- 0.3%) | 279,992  (2.7% +- 0.6%) | 153,191  (3.2% +- 1%) | .002 |
| Trauma | 24,211,362  (17.8% +- 1.7%) | 349,182  (14% +- 3.3%) | 1,540,454  (11.1% +- 1.6%) | 6,840,787  (12.1% +- 0.9%) | 12,324,837  (25.8% +- 2%) | 2,219,068  (21.1% +- 3%) | 937,034  (19.7% +- 6.4%) | < .001 |

*COPD,* chronic obstructive pulmonary disease.

**Appendix Table 2:** Resource utilization according to acuity level in 2009.

| **Variable** | **Total** (136,072,130) | **Immediate** (2,493,833) | **Emergent** (13,876,131) | **Urgent** (56,654,302) | **Semiurgent** (47,766,622) | **Nonurgent** (10,531,778) | **No triage** (4,749,464) | **p** |
| --- | --- | --- | --- | --- | --- | --- | --- | --- |
| Length of visit | 206.56 (+- 4.9) | 216.56 (+- 23.27) | 272.95 (+- 12.14) | 242.23 (+- 5.4) | 163.48 (+- 4.74) | 142.97 (+- 5.42) | 155.83 (+- 13.77) | < .001 |
| Number of procedures |  |  |  |  |  |  |  | < .001 |
| 0 | 69,781,143  (53.4% +- 4.8%) | 1,039,369  (43.4% +- 8.2%) | 5,118,171  (38.7% +- 4.3%) | 24,845,044  (45.8% +- 3.2%) | 29,308,365  (63.6% +- 4.9%) | 6,929,107  (69.1% +- 6.8%) | 2,541,087  (54.4% +- 18.8%) |  |
| 1 | 50,527,561  (38.7% +- 3.6%) | 931,543  (38.9% +- 8.6%) | 6,291,989  (47.6% +- 4.2%) | 24,389,016  (45% +- 3.4%) | 14,467,482  (31.4% +- 2.5%) | 2,669,294  (26.6% +- 3.9%) | 1,778,237  (38.1% +- 12.7%) |  |
| 2 | 8,659,576  (6.6% +- 1.1%) | 248,476  (10.4% +- 3.3%) | 1,405,732  (10.6% +- 1.6%) | 4,322,073  (8% +- 1.1%) | 2,061,062  (4.5% +- 0.6%) | 336,673  (3.4% +- 1%) | 285,560  (6.1% +- 3.3%) |  |
| 3 | 1,467,413  (1.1% +- 0.3%) | 132,097  (5.5% +- 1.6%) | 360,997  (2.7% +- 0.6%) | 600,535  (1.1% +- 0.2%) | 238,754  (0.5% +- 0.1%) | 73,143  (0.7% +- 0.3%) | 61,887  (1.3% +- 0.7%) |  |
| 4 | 171,409  (0.1% +- 0.1%) | 26,643  (1.1% +- 0.7%) | 46,813  (0.4% +- 0.3%) | 63,919  (0.1% +- 0%) | 15,361  (0% +- 0%) | 15,525  (0.2% +- 0.1%) | 3,148  (0.1% +- 0.1%) |  |
| 5 | 40,738  (0% +- 0%) | 16,191  (0.7% +- 0.4%) | 7,533  (0.1% +- 0.1%) | 7,018  (0% +- 0%) | 9,996  (0% +- 0%) | 0  (0% +- 0%) | 0  (0% +- 0%) |  |
| Number of diagnostics | 2.88 (+- 0.1) | 3.68 (+- 0.34) | 4.99 (+- 0.27) | 3.79 (+- 0.13) | 1.54 (+- 0.09) | 1.12 (+- 0.12) | 2.75 (+- 0.29) | < .001 |
| Number of medications given in ED | 1.26 (+- 0.04) | 1.74 (+- 0.22) | 1.77 (+- 0.08) | 1.49 (+- 0.05) | 0.94 (+- 0.04) | 0.71 (+- 0.04) | 1.05 (+- 0.13) | < .001 |
| Return for appointment as needed | 47,393,066  (34.8% +- 3.6%) | 642,177  (25.8% +- 7.8%) | 3,637,157  (26.2% +- 3.2%) | 19,361,021  (34.2% +- 2.9%) | 18,854,150  (39.5% +- 3.8%) | 3,633,715  (34.5% +- 3.7%) | 1,264,846  (26.6% +- 10%) | < .001 |
| Return/refer to physician/ clinic for follow up | 85,617,521  (62.9% +- 5.7%) | 1,305,002  (52.3% +- 12.8%) | 6,675,938  (48.1% +- 4.9%) | 35,258,895  (62.2% +- 4.6%) | 32,688,588  (68.4% +- 5.1%) | 6,676,723  (63.4% +- 6.8%) | 3,012,375  (63.4% +- 22.4%) | < .001 |
| Hospital admission | 17,118,576  (12.6% +- 1.4%) | 526,221  (21.1% +- 3%) | 4,518,997  (32.6% +- 3.7%) | 9,255,141  (16.3% +- 1.5%) | 2,170,040  (4.5% +- 0.6%) | 324,124  (3.1% +- 0.5%) | 324,053  (6.8% +- 2.3%) | < .001 |
| Admission to observation unit, then hospitalized | 849,035  (0.6% +- 0.2%) | 10,296  (0.4% +- 0.2%) | 238,440  (1.7% +- 0.7%) | 491,247  (0.9% +- 0.2%) | 76,877  (0.2% +- 0.1%) | 13,893  (0.1% +- 0.1%) | 18,282  (0.4% +- 0.2%) | < .001 |
| Dead on arrival | 57,547  (0% +- 0%) | 54,363  (2.2% +- 0.8%) | 0  (0% +- 0%) | 3,184  (0% +- 0%) | 0  (0% +- 0%) | 0  (0% +- 0%) | 0  (0% +- 0%) | .02 |
| Died in ED | 154,199  (0.1% +- 0.1%) | 66,906  (2.7% +- 0.7%) | 27,064  (0.2% +- 0.1%) | 26,393  (0% +- 0%) | 16,772  (0% +- 0%) | 9,764  (0.1% +- 0.1%) | 7,300  (0.2% +- 0.1%) | < .001 |
| Transfer to other hospital | 1,898,990  (1.4% +- 0.3%) | 82,240  (3.3% +- 1.1%) | 382,109  (2.8% +- 0.4%) | 787,317  (1.4% +- 0.2%) | 395,771  (0.8% +- 0.2%) | 121,051  (1.1% +- 0.6%) | 130,502  (2.7% +- 1%) | < .001 |

*ED,* emergency department.

**Appendix Table 3:** Inferences for the United States regarding characteristics of study participants, chief complaints and primary diagnosis in 2010.

| **Variable** | **Total** (129,843,377) | **Immediate** (1,485,622) | **Emergent** (13,261,120) | **Urgent** (56,346,717) | **Semiurgent** (42,433,030) | **Nonurgent** (9,025,662) | **No triage** (7,291,226) | **p** |
| --- | --- | --- | --- | --- | --- | --- | --- | --- |
| Patient age in years | 36.71 (+- 0.5) | 43.95 (+- 2.54) | 44.26 (+- 1.55) | 39.97 (+- 0.63) | 31.40 (+- 0.66) | 30.76 (+- 0.84) | 34.63 (+- 1.86) | < .001 |
| Female | 71,535,194  (55.1% +- 4.6%) | 810,812  (54.6% +- 10.1%) | 7,009,624  (52.9% +- 5.2%) | 32,389,446  (57.5% +- 3.8%) | 22,747,202  (53.6% +- 3.6%) | 4,751,043  (52.6% +- 5.5%) | 3,827,067  (52.5% +- 14%) | < .001 |
| Hispanic ethnicity | 17,127,932  (13.2% +- 2.3%) | 89,239  (6% +- 1.8%) | 1,510,227  (11.4% +- 1.7%) | 7,182,183  (12.7% +- 1.7%) | 5,559,442  (13.1% +- 1.9%) | 1,252,438  (13.9% +- 2.6%) | 1,534,403  (21% +- 10.4%) | .256 |
| Race |  |  |  |  |  |  |  | .562 |
| Asian | 2,056,582  (1.8% +- 0.4%) | 20,216  (1.5% +- 0.8%) | 238,845  (2% +- 0.5%) | 931,161  (1.8% +- 0.3%) | 607,255  (1.6% +- 0.4%) | 123,227  (1.6% +- 0.4%) | 135,878  (2% +- 1.1%) |  |
| Black | 25,920,760  (22.2% +- 3.2%) | 400,031  (29.9% +- 13.6%) | 2,731,115  (22.7% +- 4.3%) | 11,306,633  (22.2% +- 2.4%) | 8,645,397  (22.7% +- 2.8%) | 1,867,631  (23.7% +- 3.5%) | 969,953  (14.6% +- 6.3%) |  |
| Other | 1,867,410  (1.6% +- 0.5%) | 22,245  (1.7% +- 0.7%) | 202,926  (1.7% +- 0.4%) | 797,285  (1.6% +- 0.5%) | 656,152  (1.7% +- 0.5%) | 103,148  (1.3% +- 0.4%) | 85,654  (1.3% +- 1%) |  |
| White | 87,025,763  (74.5% +- 6.8%) | 893,236  (66.9% +- 8.2%) | 8,871,743  (73.7% +- 7%) | 37,881,695  (74.4% +- 5%) | 28,135,041  (74% +- 5.6%) | 5,792,899  (73.4% +- 8.1%) | 5,451,149  (82.1% +- 24.6%) |  |
| Presenting level of pain | 4.97 (+- 0.06) | 4.08 (+- 0.37) | 4.35 (+- 0.13) | 4.97 (+- 0.07) | 5.19 (+- 0.09) | 4.86 (+- 0.16) | 5.08 (+- 0.15) | < .001 |
| Temperature in Fahrenheit | 98.27 (+- 0.02) | 98.10 (+- 0.08) | 98.22 (+- 0.04) | 98.28 (+- 0.02) | 98.30 (+- 0.03) | 98.17 (+- 0.03) | 98.35 (+- 0.1) | .136 |
| Heart rate | 91.79 (+- 0.41) | 93.29 (+- 2.05) | 92.05 (+- 0.9) | 91.58 (+- 0.44) | 91.92 (+- 0.53) | 91.25 (+- 0.76) | 92.51 (+- 2.06) | .893 |
| Blood pressure - Systolic | 133.26 (+- 0.31) | 135.86 (+- 2.25) | 136.51 (+- 1.04) | 134.33 (+- 0.33) | 131.17 (+- 0.39) | 130.82 (+- 0.66) | 132.83 (+- 1.42) | < .001 |
| Blood pressure - Diastolic | 77.73 (+- 0.23) | 77.63 (+- 1.22) | 78.46 (+- 0.65) | 77.90 (+- 0.25) | 77.23 (+- 0.32) | 77.46 (+- 0.5) | 78.22 (+- 0.8) | .235 |
| Respiratory rate | 19.64 (+- 0.11) | 20.46 (+- 0.81) | 20.29 (+- 0.24) | 19.55 (+- 0.11) | 19.47 (+- 0.14) | 19.30 (+- 0.2) | 20.42 (+- 0.56) | .028 |
| Pulse oximetry (percent) | 97.21 (+- 0.24) | 94.77 (+- 1.04) | 96.62 (+- 0.24) | 97.23 (+- 0.2) | 97.44 (+- 0.29) | 97.09 (+- 0.79) | 97.48 (+- 0.24) | .069 |
| Waiting time to see physician | 50.70 (+- 1.91) | 30.57 (+- 4.17) | 43.43 (+- 2.9) | 54.75 (+- 2.25) | 52.12 (+- 2.44) | 47.44 (+- 2.89) | 33.51 (+- 4.7) | < .001 |
| Visit reason |  |  |  |  |  |  |  |  |
| Shortness of breath | 3,463,828  (2.7% +- 0.4%) | 101,360  (6.8% +- 1.7%) | 849,457  (6.4% +- 0.7%) | 1,855,193  (3.3% +- 0.3%) | 434,811  (1% +- 0.2%) | 50,423  (0.6% +- 0.2%) | 172,584  (2.4% +- 0.9%) | < .001 |
| Chest pain | 7,006,540  (5.4% +- 0.6%) | 124,055  (8.4% +- 2.1%) | 1,970,017  (14.9% +- 1.6%) | 3,604,757  (6.4% +- 0.5%) | 822,942  (1.9% +- 0.2%) | 137,965  (1.5% +- 0.3%) | 346,804  (4.8% +- 1.5%) | < .001 |
| Abdominal pain | 10,416,899  (8% +- 0.8%) | 62,826  (4.2% +- 1.6%) | 786,829  (5.9% +- 1.1%) | 7,106,389  (12.6% +- 0.9%) | 1,684,450  (4% +- 0.4%) | 276,666  (3.1% +- 0.7%) | 499,739  (6.9% +- 1.9%) | < .001 |
| Dizziness | 2,071,997  (1.6% +- 0.2%) | 2,996  (0.2% +- 0.1%) | 309,675  (2.3% +- 0.3%) | 1,172,827  (2.1% +- 0.2%) | 404,584  (1% +- 0.1%) | 35,506  (0.4% +- 0.1%) | 146,409  (2% +- 0.6%) | < .001 |
| Nausea | 4,646,375  (3.6% +- 0.4%) | 26,319  (1.8% +- 1.1%) | 285,907  (2.2% +- 0.4%) | 2,923,126  (5.2% +- 0.4%) | 1,036,407  (2.4% +- 0.3%) | 137,035  (1.5% +- 0.4%) | 237,581  (3.3% +- 1%) | < .001 |
| Shoulder pain | 1,308,184  (1% +- 0.2%) | 6,768  (0.5% +- 0.3%) | 70,089  (0.5% +- 0.2%) | 435,720  (0.8% +- 0.1%) | 621,862  (1.5% +- 0.2%) | 95,915  (1.1% +- 0.3%) | 77,830  (1.1% +- 0.5%) | .001 |
| Back pain | 3,510,459  (2.7% +- 0.3%) | 23,613  (1.6% +- 0.8%) | 175,131  (1.3% +- 0.3%) | 1,175,940  (2.1% +- 0.2%) | 1,604,830  (3.8% +- 0.3%) | 350,827  (3.9% +- 0.7%) | 180,118  (2.5% +- 0.7%) | < .001 |
| Diagnosis |  |  |  |  |  |  |  |  |
| Trauma | 6,533,767  (5% +- 0.7%) | 36,564  (2.5% +- 0.8%) | 332,101  (2.5% +- 0.6%) | 1,950,105  (3.5% +- 0.4%) | 3,156,384  (7.4% +- 0.7%) | 594,549  (6.6% +- 1.1%) | 464,064  (6.4% +- 2.1%) | < .001 |
| Fever | 4,992,272  (3.8% +- 0.5%) | 13,744  (0.9% +- 0.5%) | 358,578  (2.7% +- 0.5%) | 2,151,378  (3.8% +- 0.4%) | 1,868,688  (4.4% +- 0.5%) | 236,268  (2.6% +- 0.5%) | 363,616  (5% +- 1.7%) | .001 |
| Cough | 3,441,374  (2.7% +- 0.4%) | 1,996  (0.1% +- 0.1%) | 202,912  (1.5% +- 0.3%) | 1,369,742  (2.4% +- 0.3%) | 1,369,093  (3.2% +- 0.3%) | 308,477  (3.4% +- 0.7%) | 189,154  (2.6% +- 0.9%) | < .001 |
| Nonspecified chest pain | 4,768,543  (3.7% +- 0.4%) | 77,573  (5.2% +- 1.7%) | 1,433,948  (10.8% +- 1.3%) | 2,562,205  (4.5% +- 0.4%) | 415,482  (1% +- 0.2%) | 88,936  (1% +- 0.3%) | 190,399  (2.6% +- 1%) | < .001 |
| Cardiac arrest and ventricular fibrillation | 219,138  (0.2% +- 0.1%) | 86,557  (5.8% +- 1.6%) | 88,217  (0.7% +- 0.2%) | 40,251  (0.1% +- 0%) | 0  (0% +- 0%) | 644  (0% +- 0%) | 3,469  (0% +- 0%) | < .001 |
| Arrhythmias | 904,068  (0.7% +- 0.1%) | 112,781  (7.6% +- 1.7%) | 337,695  (2.5% +- 0.5%) | 367,722  (0.7% +- 0.1%) | 53,548  (0.1% +- 0.1%) | 644  (0% +- 0%) | 31,678  (0.4% +- 0.2%) | < .001 |
| Congestive heart failure | 676,270  (0.5% +- 0.1%) | 60,193  (4.1% +- 1.4%) | 131,435  (1% +- 0.2%) | 421,940  (0.7% +- 0.1%) | 28,168  (0.1% +- 0%) | 2,962  (0% +- 0%) | 31,572  (0.4% +- 0.2%) | < .001 |
| Acute cerebrovascular disease | 1,637,680  (1.3% +- 0.2%) | 33,819  (2.3% +- 0.8%) | 306,741  (2.3% +- 0.3%) | 732,237  (1.3% +- 0.2%) | 392,229  (0.9% +- 0.1%) | 91,839  (1% +- 0.3%) | 80,815  (1.1% +- 0.4%) | < .001 |
| Syncope | 1,247,682  (1% +- 0.2%) | 19,680  (1.3% +- 1%) | 232,765  (1.8% +- 0.3%) | 767,085  (1.4% +- 0.1%) | 135,539  (0.3% +- 0.1%) | 34,105 (0.4% +- 0.2%) | 58,508  (0.8% +- 0.3%) | < .001 |
| Anxiety disorders | 921,772  (0.7% +- 0.1%) | 5,289  (0.4% +- 0.3%) | 124,807  (0.9% +- 0.2%) | 485,344  (0.9% +- 0.1%) | 195,010  (0.5% +- 0.1%) | 78,863  (0.9% +- 0.3%) | 32,459  (0.4% +- 0.2%) | .019 |
| Gastrointestinal disease | 16,979,240  (13.1% +- 1.2%) | 90,068  (6.1% +- 2.1%) | 1,069,882  (8.1% +- 1.2%) | 10,184,154  (18.1% +- 1.4%) | 4,011,443  (9.5% +- 0.7%) | 869,819  (9.7% +- 1.1%) | 753,874  (10.3% +- 2.9%) | < .001 |
| Altered mental status | 6,331,947  (4.9% +- 0.5%) | 62,783  (4.2% +- 1.4%) | 1,003,038  (7.6% +- 0.8%) | 3,410,652  (6.1% +- 0.5%) | 1,326,069  (3.1% +- 0.3%) | 181,180  (2% +- 0.4%) | 348,225  (4.8% +- 1.6%) | < .001 |
| Upper respiratory infections | 5,916,857  (4.6% +- 0.5%) | 5,342  (0.4% +- 0.3%) | 282,897  (2.1% +- 0.5%) | 1,924,762  (3.4% +- 0.3%) | 2,734,808  (6.4% +- 0.6%) | 598,024  (6.6% +- 0.9%) | 371,024  (5.1% +- 1.4%) | < .001 |
| Pneumonia | 1,887,957  (1.5% +- 0.2%) | 31,782  (2.1% +- 1.1%) | 264,094  (2% +- 0.3%) | 1,015,488  (1.8% +- 0.2%) | 365,901  (0.9% +- 0.2%) | 70,395  (0.8% +- 0.2%) | 140,297  (1.9% +- 0.7%) | < .001 |
| Asthma/COPD | 3,607,013  (2.8% +- 0.4%) | 41,715  (2.8% +- 1.1%) | 642,894  (4.8% +- 0.7%) | 1,709,327  (3% +- 0.3%) | 893,142  (2.1% +- 0.2%) | 109,212  (1.2% +- 0.3%) | 210,723  (2.9% +- 1%) | < .001 |
| Trauma | 23,622,301  (18.2% +- 1.7%) | 211,834  (14.3% +- 2.4%) | 1,395,106  (10.5% +- 1.6%) | 6,938,456  (12.3% +- 0.8%) | 11,533,589  (27.2% +- 1.9%) | 1,947,440  (21.6% +- 2.7%) | 1,595,876  (21.9% +- 6.3%) | < .001 |

*COPD*, chronic obstructive pulmonary disease.

**Appendix Table 4:** Resource utilization according to acuity level in 2010.

| **Variable** | **Total** (129,843,377) | **Immediate** (1,485,622) | **Emergent** (13,261,120) | **Urgent** (56,346,717) | **Semiurgent** (42,433,030) | **Nonurgent** (9,025,662) | **No triage** (7,291,226) | **p** |
| --- | --- | --- | --- | --- | --- | --- | --- | --- |
| Length of visit | 201.77 (+- 5.04) | 254.07 (+- 33.29) | 270.74 (+- 11.76) | 232.69 (+- 5.63) | 157.54 (+- 6.64) | 134.67 (+- 6.28) | 168.33 (+- 17.95) | < .001 |
| Number of procedures | 0.56 (+- 0.02) | 1.01 (+- 0.1) | 0.77 (+- 0.03) | 0.64 (+- 0.02) | 0.44 (+- 0.02) | 0.35 (+- 0.02) | 0.47 (+- 0.06) | < .001 |
| Number of diagnostics | 3.17 (+- 0.1) | 5.36 (+- 0.44) | 5.35 (+- 0.26) | 4.14 (+- 0.14) | 1.69 (+- 0.1) | 1.24 (+- 0.13) | 2.22 (+- 0.26) | < .001 |
| Number of medications given in ED | 1.39 (+- 0.04) | 2.25 (+- 0.18) | 2.01 (+- 0.09) | 1.59 (+- 0.05) | 1.07 (+- 0.05) | 0.83 (+- 0.05) | 1.04 (+- 0.15) | < .001 |
| Return for appointment as needed | 44,193,056  (34% +- 4.1%) | 343,304  (23.1% +- 5.7%) | 3,050,729  (23% +- 3.3%) | 17,739,683  (31.5% +- 2.8%) | 16,379,004  (38.6% +- 3.6%) | 3,426,528  (38% +- 5.2%) | 3,253,808  (44.6% +- 16.5%) | .004 |
| Return/refer to physician/ clinic for follow up | 81,281,219  (62.6% +- 5.5%) | 573,445  (38.6% +- 6.4%) | 6,796,110  (51.2% +- 6.1%) | 35,130,664  (62.3% +- 4.1%) | 29,167,957  (68.7% +- 5.3%) | 6,100,139  (67.6% +- 7.3%) | 3,512,904  (48.2% +- 13.4%) | < .001 |
| Hospital admission | 17,239,122  (13.3% +- 1.4%) | 543,135  (36.6% +- 7.8%) | 4,128,475  (31.1% +- 2.9%) | 9,444,487  (16.8% +- 1.3%) | 2,121,935  (5% +- 0.7%) | 451,153  (5% +- 1.3%) | 549,937  (7.5% +- 2.7%) | < .001 |
| Admission to observation unit, then hospitalized | 1,030,483  (0.8% +- 0.2%) | 27,695  (1.9% +- 0.9%) | 271,529  (2% +- 0.5%) | 534,536  (0.9% +- 0.2%) | 136,358  (0.3% +- 0.1%) | 55,405  (0.6% +- 0.3%) | 4,960  (0.1% +- 0.1%) | < .001 |
| Dead on arrival | 50,617  (0% +- 0%) | 15,080  (1% +- 0.5%) | 18,626  (0.1% +- 0.1%) | 15,414  (0% +- 0%) | 0  (0% +- 0%) | 0  (0% +- 0%) | 1,497  (0% +- 0%) | .005 |
| Died in ED | 191,168  (0.1% +- 0.1%) | 74,478  (5% +- 1.5%) | 48,640  (0.4% +- 0.1%) | 56,814  (0.1% +- 0%) | 1,891  (0% +- 0%) | 5,876  (0.1% +- 0.1%) | 3,469  (0% +- 0%) | < .001 |
| Transfer to other hospital | 2,002,659  (1.5% +- 0.3%) | 77,710  (5.2% +- 1.3%) | 422,995  (3.2% +- 0.5%) | 936,370  (1.7% +- 0.2%) | 324,982  (0.8% +- 0.1%) | 32,176  (0.4% +- 0.1%) | 208,426  (2.9% +- 0.9%) | < .001 |

*ED,* emergency department.
